# Neurological Soft Signs and Thyroid Hormones in Schizophrenia

**DOI:** 10.1192/j.eurpsy.2025.712

**Published:** 2025-08-26

**Authors:** E. M. Tsapakis, M. Treiber, S. Athanasiou, K. Chovardas, T. Kyziridis, K. N. Fountoulakis

**Affiliations:** 1 3rd Department of Psychiatry, Aristotle University of Thessaloniki, Thessaloniki, Greece; 2 Department of Psychiatry and Psychotherapy, Medical Univeristy of Vienna; 3 Comprehensive Center for Clinical Neurosciences and Mental Health (C3NMH), Medical University of Vienna, Vienna, Austria

## Abstract

**Introduction:**

Neurological soft signs (NSS) are subtle sensory and motor deficits linked to neurodevelopmental disorders, schizophrenia, and thyroid disorders (TH). TH are essential for neurodevelopment and the modulation of the proinflammatory response. Indeed, a growing body of evidence suggests that thyroid function is altered in individuals with schizophrenia spectrum disorders (SSD).

**Objectives:**

We aimed to evaluate the relationship between TH, NSS, and psychopathology in individuals with schizophrenia.

**Methods:**

Opportunistic recruitment took place at the 3^rd^ Department of Psychiatry at AHEPA University General Hospital of Thessaloniki, Greece. Inclusion criteria were an SDD diagnosis and age above 18 or below 65 years. Patients were excluded if they had a history of neurological or other somatic disorder, a history of substance abuse, an IQ estimate <70, and if they were pregnant, or on treatment with glucocorticoids and/or thyroxin. Clinical symptomatology was assessed using the Positive and Negative Syndrome Scale (PANSS), and NSS were assessed using the Neurological Evaluation Scale (NES; Greek version). Blood samples were drawn after an overnight fast to measure serum levels of TSH, fT4, and fT3. We used the t-test to compare differences between sex and the Pearson correlation to test for correlations between PANSS scores, NES scores, and TH (R statistical software version 4.3.2).

**Results:**

A total of 73 patients (31 female) with SSD were included [mean age 41.2 (SD 11.6) years]. TSH and fT4 levels were significantly higher in females, p<0.0005 and p=0.020, respectively. fT3, but not fT4 or TSH, was negatively correlated with age (r=-0.479, p<0.001). A negative correlation between NES total score and fT4 (r=-0.416, p=0.014) was only found in males. Serum fT3 levels exhibited no significant correlation with NES scores but the PANSS negative subscore was negatively associated with fT3 (r=-0.471, p<0.001).

**Conclusions:**

Our study suggests that TSH and fT4 abnormalities are more prevalent in females with SSDs. Moreover, it appears that with increasing age, the likelihood of hypothyroidism increases. Interestingly, in individuals with SSDs, lower fT4 levels predicted NSS severity but only in males, and lower fT3 levels predicted an increase in negative symptoms. Hypothyroidism has been reported to cause damage to the central nervous system and has been associated with increased apoptosis and altered expression of cerebellar neurons leading to impairment in motor function. In this sense, restoring fT3 and fT4 levels might have a positive effect on negative symptoms and NSS severity (in males), respectively. However, antipsychotic medication may affect TH levels in SSDs. Thus, future studies should examine a larger sample of drug-naïve individuals with SSDs, followed-up longitudinally in time to infer causality.

**Disclosure of Interest:**

None Declared

